# Development and evaluation of a deep incremental learning system for mammography image analysis

**DOI:** 10.1371/journal.pone.0358861

**Published:** 2026-09-25

**Authors:** Mohammad Hadi Ghahroudi, Nasrin Ahmadinejad, Vahid Changizi, Elahe Ahmadi, Zahra Mohammadmirzaei, Zahra Shayegh, Seyed Mohammad Ayyoubzadeh

**Affiliations:** 1 Health Information Management and Medical Informatics Department, School of Allied Medical Sciences, Tehran University of Medical Sciences, Tehran, Iran; 2 Advanced Diagnostic and Interventional Radiology Research Center, Imam Khomeini Hospital Complex, Tehran University of Medical Sciences, Tehran, Iran; 3 Technology of Radiology and Radiotherapy Department, School of Allied Medical Sciences, Tehran University of Medical Sciences, Tehran, Iran; University of Pisa, ITALY

## Abstract

**Introduction:**

Breast cancer is a malignant tumor that mostly stems from ductal or lobular epithelial tissues. Each year, over two million people suffer from this disease and over half a million pass away globally, ranking breast cancer the most prevalent cancer type in women and the second cause of cancer-related deaths. Periodic screening programs such as mammography have significantly improved survival via early detection. Therefore, facilitating the interpretation for this modality through Computer-Aided Diagnosis (CAD) systems can accelerate specialists’ decision-making and further raise the survival rates.

**Materials and methods:**

A CAD web-based system was designed through Unified Modeling Language (UML) according to the three-tier architecture. The design was implemented using C# programming language and the ASP.NET 8 framework along with the Microsoft Structured Query Language (MSSQL) database. The ViTGEMC deep ensemble model with Incremental Learning (IL) capabilities was incorporated as the prediction engine. Moreover, the system evaluation was conducted in two phases: a multi-reader and multi-case experiment to appraise the system’s assistance effect, and the user experience assessment via the User Experience Questionnaire (UEQ).

**Results:**

The impact of software predictions was evaluated in an interventional test with the participation of four radiology specialists and 40 dual-view examinations, which yielded a 19.5% reduction in interpretation time (404.9 ± 104.2 vs. 325.8 ± 80.3 seconds, *p* = 0.0203) while maintaining accuracy (0.769 ± 0.024 vs. 0.800 ± 0.147, *p* = 0.6560). The usability assessment was further conducted by 10 experts in medicine, radiology, and health information technology fields, in which the system achieved scores of 1.4 to 2.3 across the six UEQ aspects of user experience.

**Conclusion:**

The CAD software provides a convenient and enhanced environment which can aid radiologists in quicker diagnosis.

## Introduction

Breast cancer is a malignant neoplasm of the breast epithelial tissue with mainly ductal or lobular origin, affecting over two million people and causing the deaths of more than half a million each year worldwide [1,2]. This cancer is the most common type in women and the second leading cause of cancer death in them, behind lung cancer [3]. In Iran, breast cancer ranks first in terms of incidence and fifth in terms of mortality among other cancers, regardless of gender [4]. The average prevalence of breast cancer among Iranian women between 1998 and 2018 was estimated at 23.6%, which indicates an upward trend [5]. In 2022, the Age Standardized Incidence Rate (ASIR) and Age Standardized Mortality Rate (ASMR) were reported as 30.5 and 11.0 per 100,000 Iranian women, respectively, lower than neighboring developing countries [6]. Furthermore, male breast cancer accounts for up to 1% of all breast malignancies and may encroach beyond the areola [7].

Mortality rates remained relatively unchanged from the 1930s to the 1970s, when radical mastectomy alone was the main treatment, but significant improvements in survival emerged after countries established early detection programs in the 1990s [8], including mammography. Hence, the overall mortality rate has decreased by 44% from 1989 to 2022 [9]. An overview of nine prospective randomized clinical trials suggests that screening mammography reduces breast cancer mortality in women over 50 by one-quarter to one-fifth [10]. Nonetheless, the uneven distribution of health care facilities, the cost of the screening process, and the shortage of qualified human resources, especially in deprived areas, hinder the early detection and treatment of breast cancer [11]. Therefore, facilitating the interpretation of mammography may mitigate these challenges.

To capture a digital mammogram, the patient’s breast is compressed and X-rays pass through the tissue and reach the detector. Based on the orientation of X-ray beam and detector, there are two conventional views: Craniocaudal (CC) and Mediolateral Oblique (MLO). The compression paddle is pressed in an inferior-superior direction in CC, and at a 40–60 degree angle in MLO to capture the pectoral muscle and axilla as much as possible [12]. While interpreting a mammogram, the radiologist searches for two categories of lesions: calcifications (small, high-contrast, and with a specific distribution and shape pattern which may be indicative of malignancy) and soft tissue findings (masses with varied shapes and margins, architectural distortion, and asymmetry) [13]. Growth in these findings is the main criterion for suspicion, so comparison with previous images is essential to increase sensitivity and accuracy. The Breast Imaging-Reporting and Data System (BI-RADS) [14] has standardized the process of analysis and reporting in breast imaging and expedited monitoring of outcomes. The patient’s status is organized into seven categories based on the morphological manifestations of the lesions. These include BI-RADS 0 (incomplete, requires previous or further imaging), BI-RADS 1 (negative, normal findings), BI-RADS 2 (benign findings), BI-RADS 3 (probably benign findings with 0–2% chance of malignancy), BI-RADS 4 (suspicious findings with 2–95% chance of malignancy), BI-RADS 5 (highly suggestive findings with more than 95% chance of malignancy), and BI-RADS 6 (findings with biopsy-proven malignancy). Further, BI-RADS 4 includes three subcategories a, b, and c, which represent a probability of 2–10%, 10–50%, and 50–95% of malignancy, respectively. One study demonstrated that a radiologist with at least 5 years of experience had a sensitivity of 97%, specificity of 64.5%, Positive Predictive Value (PPV) of 89%, Negative Predictive Value (NPV) of 90.9%, and accuracy of 89.3% for the diagnosis of breast cancer based on mammography images [15]. Although radiologists’ improved performance in mammography interpretation is not vividly linked to a higher number of viewed mammograms or experience, the volume of interpreted images and expertise in breast imaging may improve sensitivity, specificity, and detection rates by setting a hypothetical threshold for considering a mammogram positive [15,16].

Considering the occurrence of lesions with distinct characteristics and outcomes, a Decision Support System (DSS) can assist the radiologists in improving their diagnostic performance. In the era of rapid advances in computer-aided diagnosis, the need for innovative solutions in the medical field has become more apparent than ever. Yet one of the challenges of intelligent systems is the continuous learning and adaptation as more training data emerge. It can be defined as the capacity of a model to incorporate new training data continuously without requiring full retraining from scratch. This approach enables a DSS to evolve over time, integrating newly acquired cases and learning new patterns while preserving previously learned knowledge. A previous study by our research team aimed to present an innovative approach for the continuous training of an ensemble model in the case of the emergence of new datasets in the future. Therefore, an ensemble of Deep Convolutional Neural Networks (DCNN) called Vision Transformer Guided Ensemble of Mammogram Classifiers (ViTGEMC) was developed. Besides incremental capability, this ensemble approach allows both parallel and sequential independent training of multiple DCNNs, providing higher flexibility. Consequently, this study aims at the deployment and evaluation of ViTGEMC as a web-based platform capable of classifying breast disease. Here, the model processes the CC and MLO mammograms uploaded by the specialist and predicts the probability of malignancy and visualizes the Regions Of Interest (ROI).

Recent studies have developed Incremental Learning (IL) solutions across different aspects and modalities, such as domain-IL for chest X-ray [17] and skin lesion [18] classification, class-IL [19] and domain-IL [20] for whole slide image classification, class-IL for ultrasound image classification [21], class-IL and domain-IL for brain MRI segmentation [22] and domain-IL for cardiac MRI segmentation [23]. Nonetheless, the application of IL to the analysis of mammograms as a CAD system, along with its usability evaluation, appeared to be inadequately explored. Hence, this study is significant in terms of developing a CAD tool based on the IL paradigm for mammography, usability evaluation, and measuring the system’s impact on the radiologists’ accuracy and speed in diagnosing breast malignancies as a multi-reader and multi-case experiment.

## Related works

There are recent noteworthy contributions studying the role of Artificial Intelligence (AI) systems in the interpretation process across various breast imaging modalities or evaluating their impact on efficiency and performance.

Focusing on commercial systems, Pacilè et al. [24] evaluated the MammoScreen [25], which processes Digital Breast Tomosynthesis (DBT) and mammography images. The study involved 25 radiologists with 2–35 years of experience to assess the system’s impact on their performance, reading time, and perceived fatigue. On average, the system’s assistance yielded a 26% increase in accuracy, a 7.4% increase in Area Under the Receiver Operating Characteristic Curve (AUC), an 8% increase in cancer detection rate, and a 24% decrease in reading time and fatigue. Furthermore, Vasilev et al. [26] conducted a multistage evaluation of the Celsus Mammography AI model [27], which is based on Faster-RCNN [28] with ResNeSt [29] backbone. The software provides malignancy probability and ROIs per mammogram view. The testing procedure comprises integration into the Picture Archiving and Communication System (PACS), functional and clinical testing by an expert radiologist to meet the baseline requirements, calibration testing to assess the model’s performance against ground truth, monitoring of technical defects, clinical monitoring of model predictions by a radiologist, feedback collection, and model updating by the developer. The proposed lifecycle-based methodology resulted in 24.7%, 15.6%, 37.1%, and 10.7% increases in AUC, accuracy, sensitivity, and specificity, respectively. Another commercial software, Lunit INSIGHT MMG [30], was evaluated by Lee et al. [31] in terms of radiologists’ performance and speed improvement in reading mammograms. The experiment encompassed 10 specialists and 200 patients. The findings implied an increase in sensitivity and AUC in the assisted condition, and no change in specificity. Further, the reading time of breast specialist radiologists decreased while it increased for general radiologists. Moreover, the Transpara [32,33] DSS was evaluated by Winkel et al. [34] for analyzing DBT images. In this experiment, 240 bilateral DBT exams were interpreted by 18 experts in AI-assisted and unassisted reading conditions, demonstrating a significant 3% increase in AUC and 12% in speed. Similarly, the same DBT analyzer software was assessed by Pinto et al. [35] involving 14 radiologists and 198 DBT exams. Despite a significant increase in AUC and sensitivity, no significant improvement in specificity and reading speed was observed by the researchers.

On the other hand, Tan et al. [36] implemented a dual-view and multi-level CNN performing at patch and breast levels. The performance of 10 radiologists was assessed with and without the model’s predictions. They were asked to provide BI-RADS and malignancy probability scores given a total of 1,302 dual-view images. The presence of the model’s assistance significantly increased average radiologist diagnostic ability in terms of AUC and sensitivity by 0.018 and 0.062, respectively. Qian et al. [37] proposed a multimodal approach incorporating dual-view mammograms, orthogonal-view ultrasound, and clinical metadata, which performed close to the invasive pathology examination. With an AUC of 0.825, the model mostly outperformed each individual assessment of five experienced radiologists. Another multimodal network integrating both mammography and ultrasound images was introduced by Xu et al. [38]. The model improved the diagnostic performance of four radiologists with 3–10 years of experience and increased the AUC by 0.176, the specificity by 20.9%, and cut down redundant biopsies by 20.35%. Similarly, Tian et al. [39] compared a model based on dual-branch ResNet50 with four radiologists for the BI-RADS 3 lesion classification task given ultrasound images. The researchers utilized two CycleGAN [40] networks to generate and augment data. The system was superior in terms of AUC and sensitivity, but with lower specificity in detecting malignant lesions.

Moreover, there are a considerable number of DL-based commercial systems in the field of mammography analysis. CommaMed introduced a diagnostic support system called GITAI [41] which was trained on more than 400,000 mammograms. The software highlights the suspicious area with an oval border and predicts the cancer risk percentage, with values above 50% being equivalent to BI-RADS 4, 5, and 6. The model was claimed to reduce the interpretation delay by 56% with a sensitivity, specificity, and AUC of 83.33%, 75.8%, and 88%, respectively. Another product is Breast-SlimView [42] software from Hera-MI which identifies and removes all physiologically normal areas (vessels, glandular tissue, adipose tissue, and mammary glands) and replaces them with artificial fat. Moreover, MammoScreen [25] is another product from the Therapixel company which offers a proprietary scoring system for risk prediction per lesion and breast, consideration of prior exam, and breast density classification.

## Materials and methods

### Ethics statement

The research methodology was approved by the Institutional Ethics Committee of Tehran University of Medical Sciences, and the informed consent requirement was waived regarding the retrospective design and use of anonymized data (ethics ID: IR.TUMS.SPH.REC.1403.055).

### Data sources

This study employed three Full-Field Digital Mammography (FFDM) datasets representing distinct populations and clinical settings:

1. *Chinese Mammography Database (CMMD)* [43]: A collection of 5,202 FFDMs from 1,775 Chinese patients who underwent examination at two centers between July 2012 and January 2016. The patients were 17–87 years old. The mammograms were acquired using Senographe DS (GE Healthcare, Chicago, IL, USA) and Mammomat Inspiration (Siemens, Erlangen, Germany) systems. This dataset lacks BI-RADS assessment and the samples are classified as benign or malignant.
2. *VinDr-Mammo* [44]: This database encompasses 20,000 FFDMs from 5,000 patients of Vietnamese ethnicity. The examinations were performed in the Hospital 108 and Hanoi Medical University Hospital in Vietnam between 2018 and 2020. The patients aged between 14 and 88 years old. The mammography systems which captured images include Giotto Class (IMS Giotto, Bologna, Italy), Giotto Image 3DL (IMS Giotto, Bologna, Italy), Mammomat Inspiration (Siemens, Erlangen, Germany), and Planmed Nuance (Planmed Oy, Helsinki, Finland). This database contains overall BI-RADS scores and density levels from A to D per breast.
3. Internal dataset: This dataset was gathered by the research team and comprises 5,766 FFDMs captured from Persian women referred to the Cancer Institute of Imam Khomeini Hospital Complex (IKHC), Tehran, Iran, from June 18, 2023 to August 12, 2024. The participants’ ages ranged from 25 to 89 years old. The mammograms were captured using FDR‑3000AWS (Fujifilm, Tokyo, Japan) and Selenia Dimensions (Hologic, Marlborough, MA, USA) systems. The patients’ images and records were accessed through the department’s PACS on August 19, 2024. The BI-RADS scores were extracted from the document associated with each mammogram, and other information was discarded to ensure the inability to identify individual participants after data collection.

**Fig 1:**
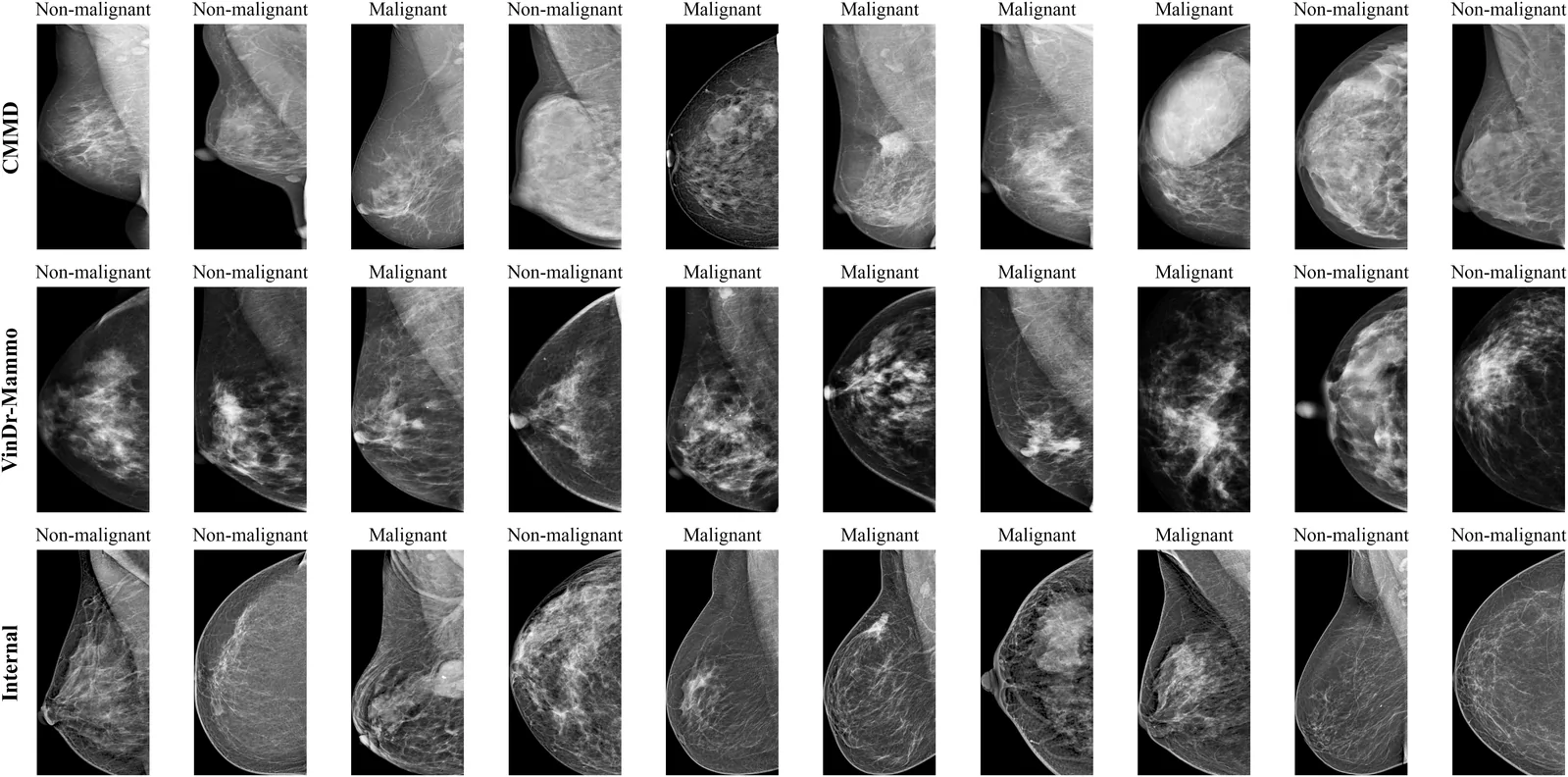
Sample mammography images from the utilized datasets. The samples represent variability in breast tissue appearance, positioning, and image characteristics across sources and classes.

Fig 1 presents malignant and non-malignant sample mammograms from each dataset.

The provided BI-RADS scores were transformed into binary labels by considering categories 1, 2, 3, and 4a as non-malignant, and 4b, 4c, 5, and 6 as malignant. Although this conversion allows for a common binary target, it does not make the datasets completely homogeneous as there are still differences in terms of label provenance, population characteristics, acquisition devices, imaging protocols and class distributions. Therefore, instead of merging the datasets into one training set, we trained a classifier for each dataset and transferred an input to the dataset-specific classifier that its training data was most similar to the input. Table 1 provides the number of malignant and non-malignant mammograms in each dataset along with the percentage of malignant cases. The three datasets comprised a total of 30,968 mammography images in Digital Imaging and Communications in Medicine (DICOM) format, which were first permuted and split into 80% training and 20% test sets, and then the training set was randomly subdivided into 90% training and 10% validation sets three times. The augmentation operations included MixUp [45], random horizontal and vertical flipping, and random resized cropping, performed for the data batch during each training iteration.

**Table 1:** Number of mammograms per class in each dataset and the proportion of positive samples.

Dataset	Malignant	Non-Malignant	Total	Positive percentage
VinDr-Mammo	988	19,012	20,000	4.94%
CMMD	2,632	2,570	5,202	50.59%
Internal	316	5,450	5,766	5.48%
Total	3,936	27,032	30,968	12.71%

### Incremental learning model

**Fig 2:**
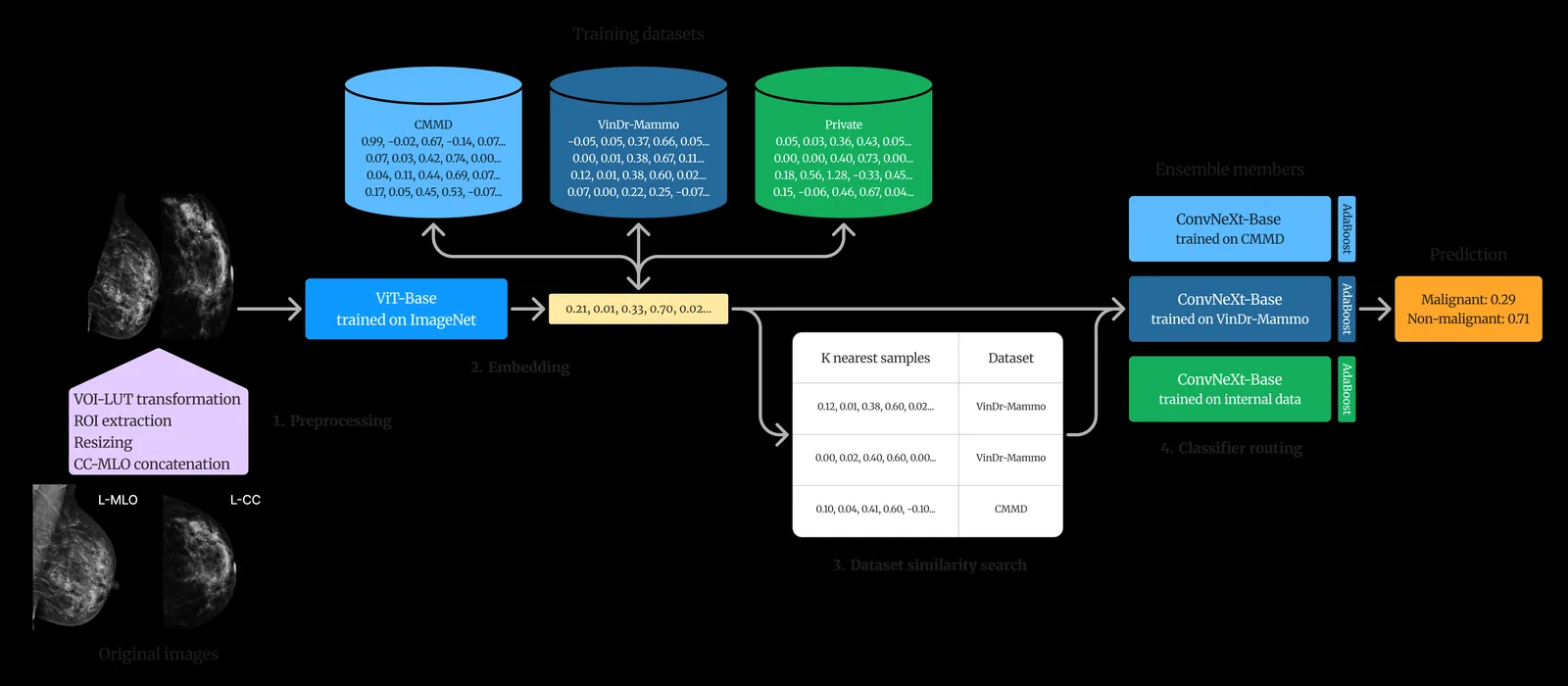
Architecture of the ViTGEMC incremental mammography classifier. The architecture extracts embeddings from dual-view craniocaudal (CC) and mediolateral oblique (MLO) images, identifies the most similar training set via cosine similarity, and routes the input to the corresponding ConvNeXt-based classifier.

The previously developed and evaluated deep ensemble model, ViTGEMC, is utilized as the prediction engine. As presented by Fig 2 block diagram, the mechanism of this model consists of:

1. *Preprocessing*: With the aim of contrast enhancement and ROI extraction, this step included applying Volume of Interest Lookup Table (VOI-LUT) values, Min-Max normalization, extraction of the breast ROI by finding the largest connected component from the binary image, resizing to 1,024 pixels in height by 512 pixels in width through bilinear interpolation, and concatenating the CC and MLO views along the y-axis. The binary image was acquired using a fixed threshold and the largest connected component is extracted using 8-way connectivity. This isolated the single largest continuous region of tissue (the breast itself), discarding any small unconnected artifacts or noise. The average resolution of original images varied across the datasets from 2,294 × 1,914–4,794 × 3,801 pixels. The monochrome mammogram was converted to a three-channel image by replicating the values across the three channels.
2. *Embedding*: For this task, a ViT-Base [46] network pretrained on ImageNet [47] with its classifier head removed extracts an embedding vector of size 768 from each dual-view CC-MLO image.
3. *Dataset similarity search*: This step operates based on the similarity between the input image and the training images of each ensemble member. The input image is assigned to the most similar training dataset by calculating the cosine similarity of its vector with each of the training image vectors and finding the k nearest neighbors (k = 9).
4. *Classifier routing*: The dataset associated with the most of the retrieved neighbors is assigned to the input image. Finally, the ensemble member trained on that dataset is chosen for malignant vs non-malignant classification and the input image is presented to it.

This approach allows both the parallel and incremental training of independent DCNNs, which can be heterogeneous in architecture. In the case of ViTGEMC, each ensemble member is a ConvNeXt-Base [48] network followed by an AdaBoost [49] model as the classifier head, which was trained on a separate specific dataset, simulating the incremental addition of new data. These data were FFDM images from the CMMD, VinDr-Mammo, and internal datasets.

The IL performance of ViTGEMC was validated by simulating the progressive addition of data using the three datasets and comparing it against a non-IL baseline; hence, this process comprised three incremental updates. Ultimately, the proposed approach reduced the total training time and achieved 96.2% accuracy, 79.0% sensitivity, 98.1% specificity, 82.7% precision, 97.6% NPV, and an AUC of 0.964. At each incremental updating stage, the resulting model was evaluated independently on the held-out test set of each of the three datasets to assess both performance on newly incorporated data and retention of performance on previously learned datasets (Table 2).

**Table 2:** Performance of ViTGEMC across the three incremental training updates. The model was evaluated separately on the CMMD, VinDr-Mammo, and internal test sets after each incremental update.

Train set	Test set	Accuracy	Sensitivity	Specificity	Precision	NPV^*^	AUC
CMMD	CMMD	0.826	0.840	0.811	0.820	0.832	0.883
	VinDr	0.571	0.781	0.567	0.029	0.994	0.802
	Internal	0.953	0.450	0.971	0.360	0.980	0.838
CMMD + VinDr	CMMD	0.815	0.802	0.829	0.827	0.804	0.860
	VinDr	0.995	0.781	0.998	0.893	0.996	0.977
	Internal	0.957	0.250	0.982	0.333	0.973	0.728
CMMD + VinDr + Internal	CMMD	0.813	0.798	0.829	0.827	0.801	0.860
	VinDr	0.995	0.781	0.998	0.893	0.996	0.977
	Internal	0.981	0.700	0.991	0.737	0.989	0.914

* NPV: Negative Predictive Value.

We further utilize the Gradient-weighted Class Activation Mapping++ (Grad-CAM++) [50] technique to generate heatmaps representing the ViTGEMC attention as a visual aid for the user. It highlights the areas of interest where the model mostly attended to during prediction. Since this heatmap does not serve as a segmentation mask, it is not independently validated against ground-truth lesion annotations in this study.

### System design and deployment

#### Back-end.

This step involves the design and implementation of the server-side software and database according to the three-tier paradigm, employing C# programming language and the ASP.NET 8 framework along with the MSSQL 2019 database. The database is built via the code-first technique and the Entity Framework technology. Furthermore, user authentication, authorization, and access levels are implemented using the Identity technology of ASP.NET.

The three-tier architecture is a widely used design paradigm which divides software into three modules: presentation tier, application logic tier, and data tier. This architecture allows for modularity and distinct development and maintenance of layers, which is especially useful in complex software systems. The presentation layer is responsible for the user experience and interface. It interacts directly with end users, presenting data and receiving input. The application logic layer acts as a mediator that processes user requests, applies logic, and communicates with the data layer. Finally, the data layer is responsible for storing and arranging data, managing database interactions, and ensuring data integrity.

#### Front-end.

At this stage, the User Interface (UI) is implemented using JavaScript, Hypertext Markup Language (HTML), and Cascading Style Sheets (CSS). Moreover, the Bootstrap library (version 5) is utilized to make page layouts responsive to various screen sizes, and the jQuery library (version 3) is used for asynchronous user input submission. DICOM medical images require special viewer software, different from regular image viewers, and hence the current step mainly involves implementing and integrating a program to view and manipulate DICOM mammography images. For this purpose, we use the Cornerstone.js library, which allows for efficient display and interaction with medical images in a web application.

### Evaluation of system’s performance and usability

The final phase includes the evaluation of the proposed system in two parts. The first part aims to understand the impact of the AI assistance on the accuracy and speed of radiologists while interpreting patients’ examinations, and the second part aims to receive the opinions and feedback of experts on the experience of working with the software.

In the first evaluation phase, the statistical environment and population are the Radiology Department of the IKHC Cancer Institute and the radiology specialists in this department, respectively. Initially, 40 patients, each having images in CC and MLO views (a total of 80 images), were randomly selected from the test subset of each of the three datasets. To include all different diagnostic groups in the sample, four patients were selected from each of the BI-RADS categories 1, 2/3, 4, and 5/6 from the internal and VinDr-Mammo datasets. Since CMMD specified benign and malignant labels only, four patients were selected from each class of this dataset. Therefore, 16, 16, and 8 patients were randomly selected from the test sets the internal, VinDr-Mammo, and CMMD datasets, respectively. After uploading the images to the software, half of the samples are processed by ViTGEMC and the other half are retained without model analysis. Then, four radiologists working at the Cancer Institute with at least 4 years of experience are selected via convenience sampling, and after getting fully familiar with the system’s environment, they begin interpreting patient images. Further in this selection process, it is ensured that the radiologists have not previously viewed any of the sampled images. Before the reading experiment, the participating radiologists underwent a five-minute orientation session conducted by the system developer. The session included a demonstration of the software features and instructions on how to use the DICOM viewer. The readers are asked to enter the final diagnosis as BI-RADS category considering only the two image projections, without the patient’s history and complementary tests. Further, they are kept unaware of the proportion of cancer cases in the population. The sampled images of the internal dataset are first presented to the radiologist, followed by the VinDr-Mammo, and finally the CMMD datasets. In each dataset, samples with no AI analysis are presented before those with model prediction. The interpretation time is measured and recorded by an independent observer using a stopwatch. Timing begins when the case is opened in the viewer and stops when the radiologist enters the final BI-RADS score. The radiologists were kept unaware that image interpretation time or accuracy was our metric for comparison. At last, we assess the significance of the system’s impact on readers’ speed and performance via a paired t-test. The case lists, image identifiers, and model outputs presented to each radiologist are provided in S1–S4 Files.

**Fig 3:**
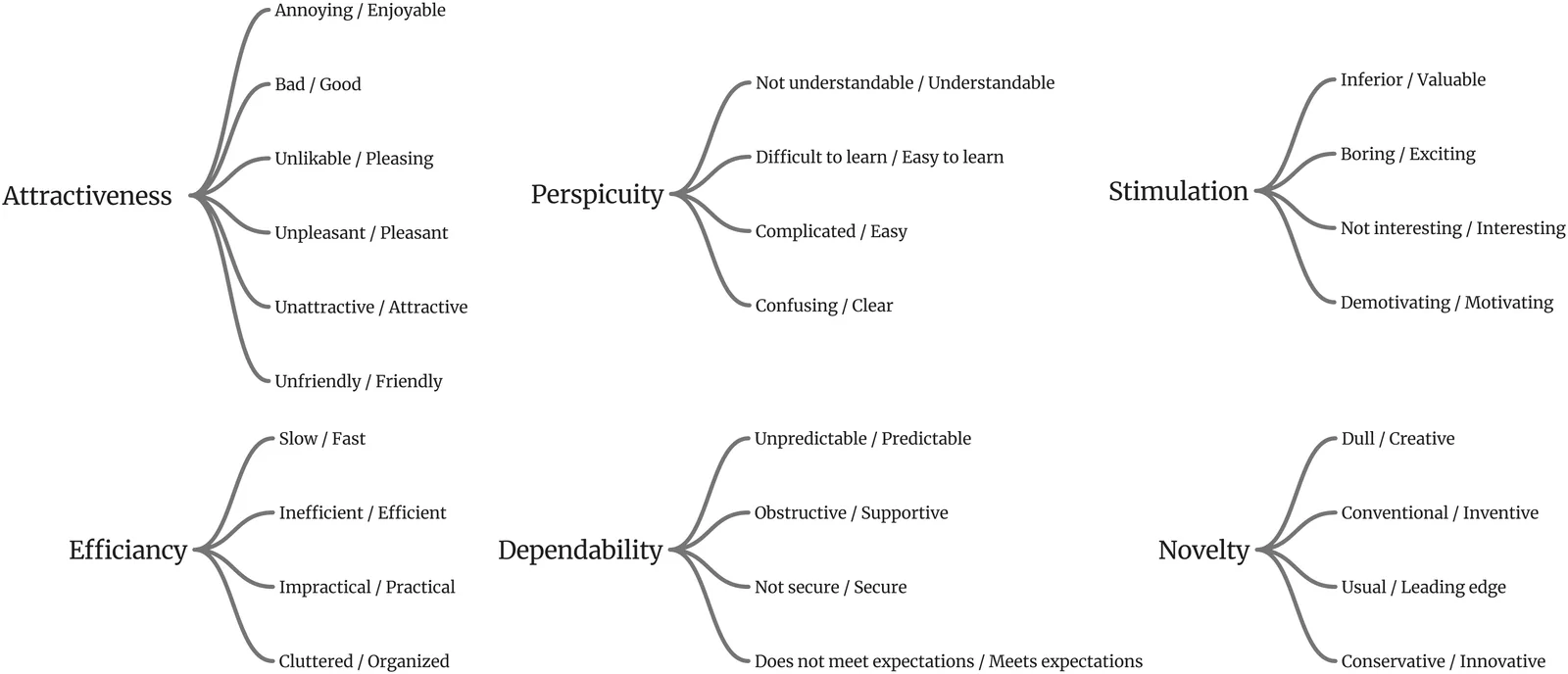
Aspects measured by the User Experience Questionnaire (UEQ). Each branch shows the opposing adjective pairs used to evaluate user perceptions from that dimension.

In the second evaluation phase, the statistical population is the faculty members of the radiology and health information management departments of Tehran University of Medical Sciences, and the selection is performed through convenience sampling. The evaluators are surveyed using the Persian version of the UEQ [51], the validity and reliability of which have been confirmed previously (Cronbach’s alpha = 0.813) [52]. Responses were analyzed using the publisher’s Excel-based UEQ Data Analysis Tool (S5 File). The UEQ questionnaire consists of 26 scales, each describing a distinct qualitative aspect of an interactive product [53]. Each scale is represented by two opposite terms on either side, between which there are 7 options to choose from. The order of attribute pairs is random, meaning half of the scales begin with a positive term and the other half with a negative term. The UEQ tool measures user experience in six aspects, each of which includes several pairs of attributes (Fig 3). The attractiveness aspect reflects the overall perception of the product by clients, indicating whether they like it or not. The perspicuity aspect includes the ease of getting familiar with the product and learning to use it. The stimulation aspect implies the extent to which clients are motivated and excited to use the product. The efficiency aspect indicates whether users can achieve their tasks without unnecessary effort. The dependability aspect measures the degree to which the user feels in control during the interaction. And the novelty aspect determines whether the product is innovative and creative or ordinary.

## Results

### System architecture

The resulting system, named Breast Imaging Novel Assistant (BINA), is a web-based software with the ability to display and manipulate mammograms, powered by the ViTGEMC incremental model as the decision support module. This software was deployed and is available at https://ai-bina.ir.

**Fig 4:**
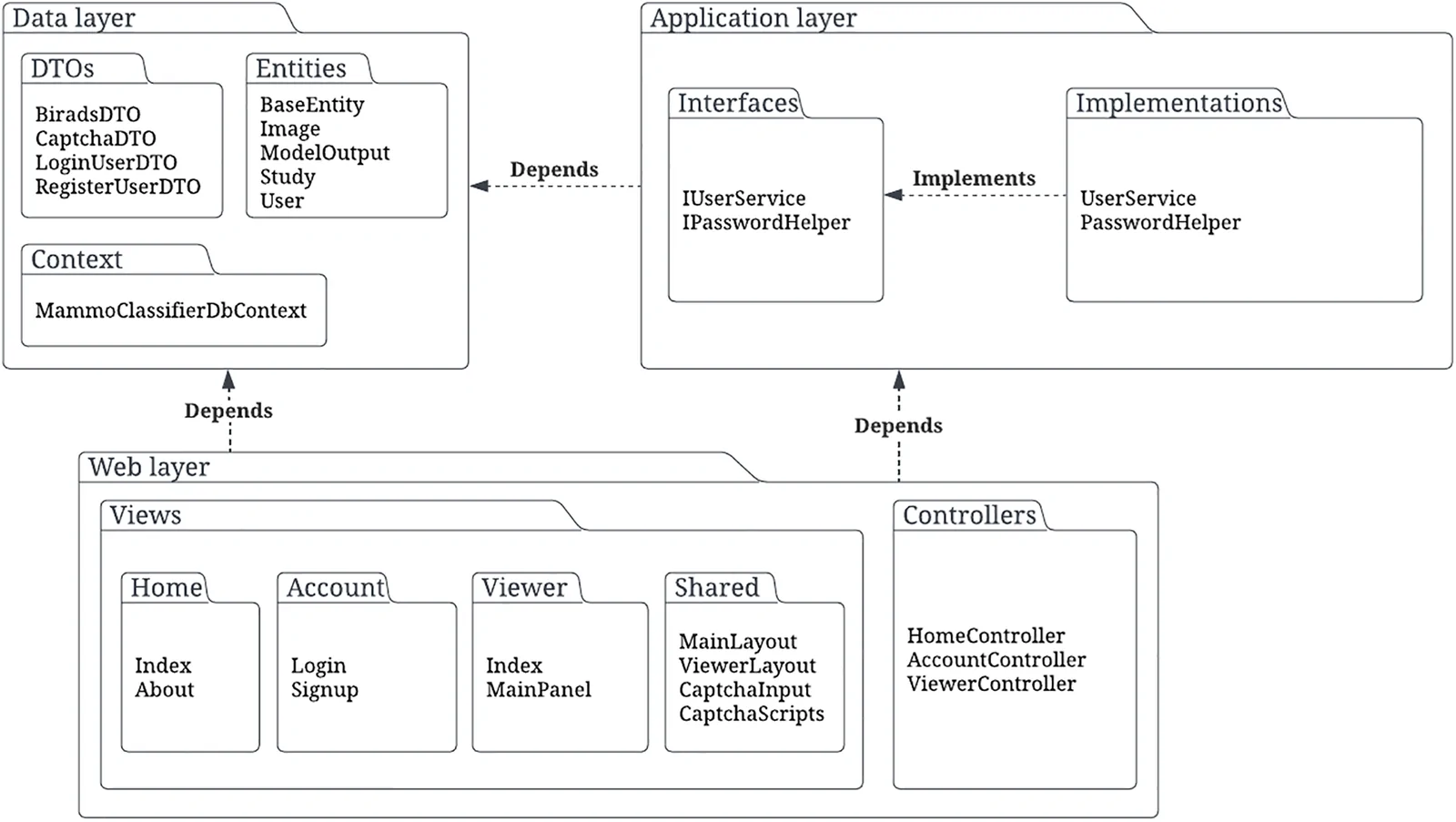
The three-tier structure of the system. This package diagram shows the relationships, dependencies, and constituent elements of the data (database entities and create/read/update/delete operations), application (business logic and services), and web (user interface and controllers) layers.

The present system is composed of three logical layers according to the three-tier architecture. The UML package diagram in Fig 4 depicts these three layers along with the classes and logical components of each, which include:

*Data layer*: responsible for managing data storage and maintaining the database connection. The entities are defined and converted into database tables via migrations in this layer. It further implements the Create, Retrieve, Update, and Delete (CRUD) operations. It includes the entity classes BaseEntity, Image, ModelOutput, Study, and User. Moreover, the Database Context (DbContext) and Data Transfer Objects (DTOs) including BiradsDTO, CaptchaDTO, LoginUserDTO, and RegisterUserDTO are implemented in this module. The DbContext class acts as a database interface and is responsible for managing connections, tracking entity modifications, and executing queries. DTOs are used to encapsulate data and transfer it between subsystems, and are responsible for information exchange between the Web and Data layers to prevent the UI from directly accessing the entity models. This approach can further reduce the load of data transferred between system components.

*Application layer*: responsible for processing user requests, applying business logic, and decision making, acting as an intermediary between the Web and Data layers. It contains services such as UserService, which implements the operations necessary for user registration and authentication.

*Web layer*: this is where the user interactions take place, and consists of the definition of UI as Views and the logic required to display them as Controllers. It further handles user input and presents the output in JavaScript Object Notation (JSON) or HTML formats. The Views are categorized in the Home, Account, Viewer, and Shared folders, which respectively incorporate pages of home, login and registration, patients list and images, and the main UI layout. Security considerations, such as authorization and access control, are defined in the Controllers section. During user registration and authentication, the session is evaluated via reCAPTCHA version 3, which identifies automatic access to the website without interrupting the user.

**Fig 5:**
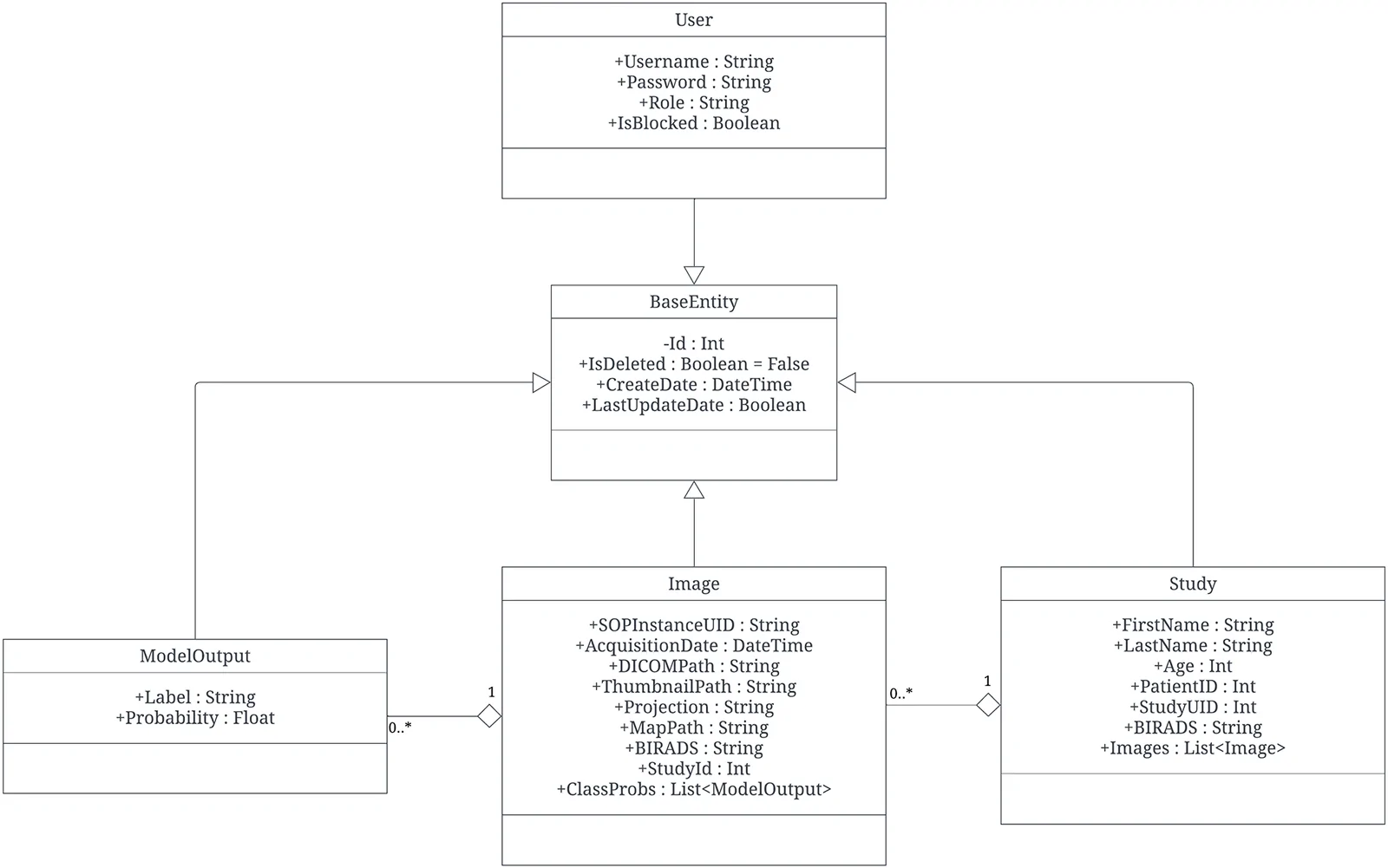
Data layer entities and their attributes. This class diagram incorporates user accounts, patient examinations (studies), mammography images, and model outputs. All entities inherit shared attributes from the BaseEntity class. Each Study is linked to several Image records, whereas each Image may have several ModelOutput entries that indicate normal or malignancy probabilities.

The UML class diagram in Fig 5 depicts the data layer entities along with their attributes and relationships. The common properties defined in the BaseEntity hold the unique identifier, deleted status, and the creation and last update dates. Hence, other entities possess these base attributes via inheritance. Furthermore, the password, username, blocked status, and role are recorded for each user. Roles include Administrator and DefaultUser, with the primary difference being the Administrator’s ability to register new users. Moreover, patient information, images, and model predictions are organized into the entities Study, Image, and ModelOutput. Each patient may possess multiple mammograms, which are available through the Images property of a Study object. Similarly, multiple model predictions for different classes can be assigned to an image object, which are accessible through the ClassProbs property.

**Fig 6:**
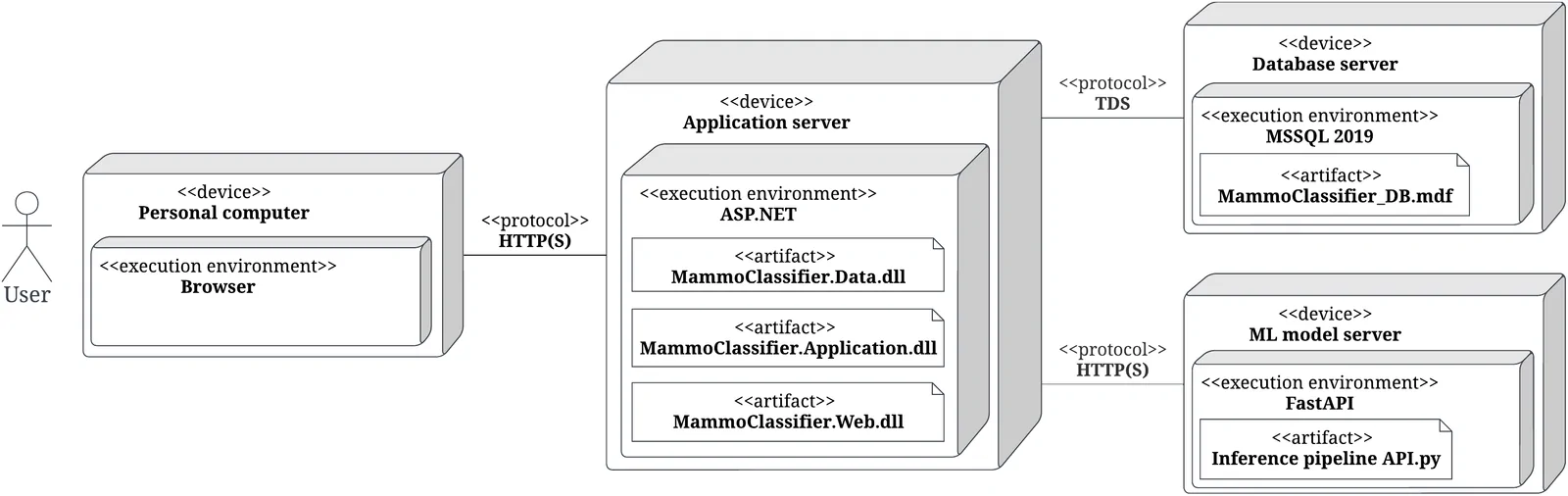
System hardware and execution environment configuration. This deployment diagram outlines machine learning, database, and application servers, as well as their communication protocols, designed to support modularity, scalability, and secure interaction during real-time analysis. Abbreviations: HTTPS, Hypertext Transfer Protocol Secure; TDS, Tabular Data Stream.

The system components were deployed across three servers, as indicated by the UML deployment diagram in Fig 6. The application server consists of three modules: the data management section (MamnoClassifier.Data), the application logic core (MamnoClassifier.Application), and the presentation section which establishes Hypertext Transfer Protocol Secure (HTTPS) communication with the web browser (MamnoClassifier.Web). The application server runtime environment is based on ASP.NET, and communication with other components is established via HTTPS and Tabular Data Stream (TDS) protocols. The database server hosts a database named MamnoClassifier_DB which operates on the MSSQL 2019 environment. Finally, the Machine Learning (ML) server stores and runs the ViTGEMC instance as a FastAPI program named Inference pipeline API.py, which processes requests and provides predictions. The hardware and software specifications of each server are presented in Table 3. The server hardware was selected to suit its tasks. The application and database servers ran on similar hardware, while the ML model server had more processing power and capacity.

**Table 3:** Hardware and software characteristics of the main system components.

Server	Hardware	Framework, Programming language (version)
Application	Processor: Intel Core i7-8550U CPU 1.80GHz 1.99GHz Memory: 16.0 GB Graphics card: NVIDIA GeForce GTX 1050	ASP.NET 8.0, C# 12.0
Database	Processor: Intel Core i7-8550U CPU 1.80GHz 1.99GHz Memory: 16.0 GB Graphics card: NVIDIA GeForce GTX 1050	MSSQL 2019, SQL (15.0.2130.3)
ML model	Processor: 12th Gen Intel Core i7-12700K 3.60GHz Memory: 32.0 GB Graphics card: NVIDIA GeForce RTX 3090	FastAPI (0.115.5), PyTorch (2.5.0), Scikit-learn (1.5.0), Python (3.10.12)

**Fig 7:**
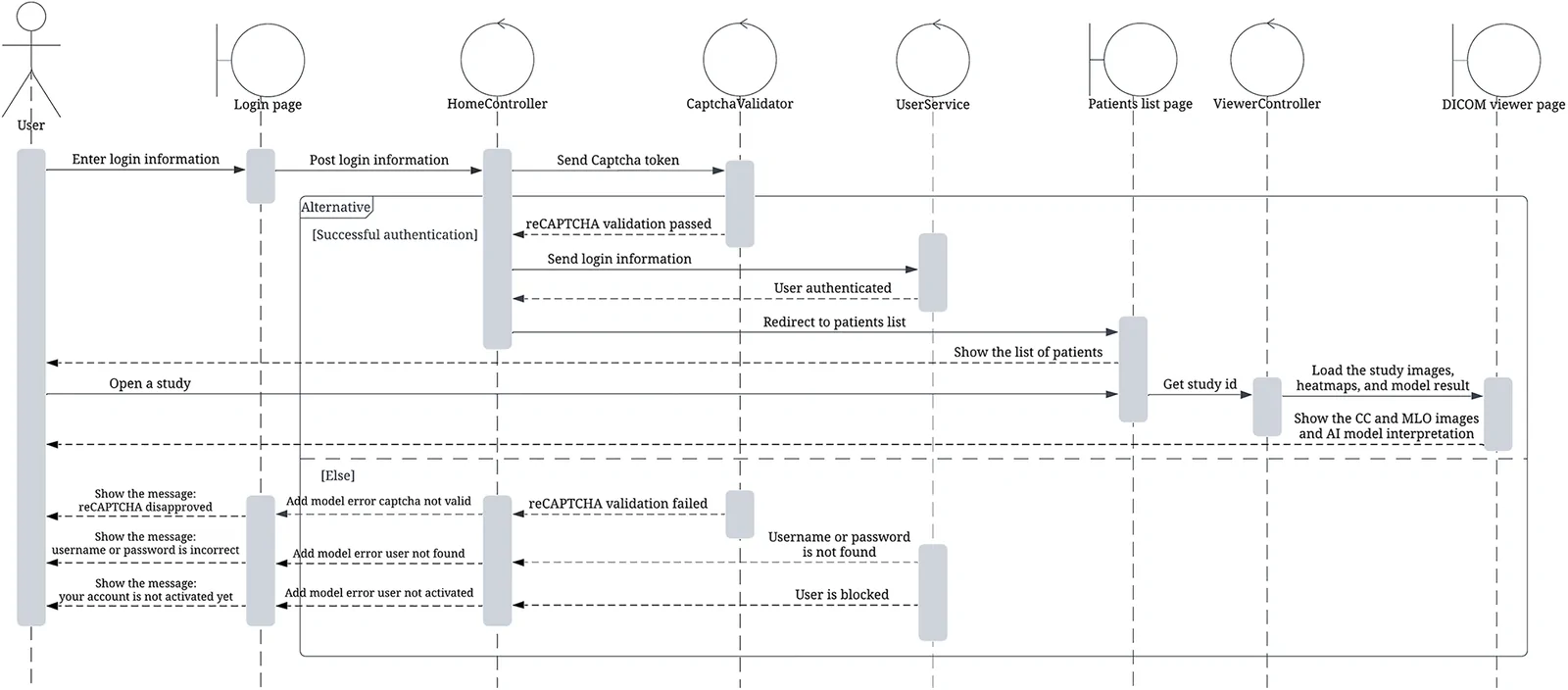
The sequence of actions in software processes. This sequence diagram depicts the end-to-end user workflow, including login, authentication, and access to patient data and model analysis. The diagram also shows alternative error paths.

An overview of the system’s workflow from user login to patient information access is depicted by the UML sequence diagram in Fig 7. The home page includes a short introduction and a button to enter the application. If the user is not authenticated, this button will redirect to the login page. A new user is registered by the admin via the signup page. The reCAPTCHA validation is performed by sending a token from the login/signup form to the server and checking via the CaptchaValidator service. If validated successfully, the UserService receives the username and password, then the password is hashed and compared with the recorded hash in the Users table of the database. In the case of a successful login, the user is granted access to the list of patients, which includes the first and last names, age, identity number (ID), and record creation date. By pressing the open button next to each patient’s record, the corresponding images can be viewed in the DICOM viewer along with the AI model analysis. At the viewer’s page, the patient’s mammograms are displayed in CC and MLO projections along with the name, age, and patient ID. Further, a text box is provided to the radiologist for entering the final BI-RADS score upon assessment. The AI model outputs include the predicted label and its probability, as well as the Grad-CAM++ heatmap overlaid on the images. The implemented viewer offers useful features for manipulating and editing DICOM images, such as contrast adjustment, moving the image, zooming, applying values of window center and width, and toggling the heatmap on or off. Moreover, navigation between patient records is made simple via the buttons placed on the right and left edges.

### System evaluation results

#### The impact of AI assistance on radiologists’ speed and performance.

In the first evaluation phase, we conducted an interventional experiment comparing the accuracy and total interpretation time of the radiologists with and without the help of the model’s analysis. Table 4 presents the system’s impact on diagnostic accuracy, which depicts an increase in average accuracy of radiologists in the presence of AI assistance. Here, the accuracy of the model alone was 90%. Likewise, the average estimated AUC values were 0.769, 0.800, and 0.900 in the human-only, AI-assisted, and AI-only scenarios, respectively. Nonetheless, this increase is not statistically significant (t(3) = 0.4927, p = 0.6560) based on the paired t-test. Therefore, the positive effect of the system assistance on the radiologists’ accuracy or AUC is not confirmed. Moreover, the impact on the time spent interpreting images by radiologists is visible in Table 5. The average interpretation time was reduced by more than one minute (19.5%), which is statistically significant (t(3) = 4.5127, p = 0.0203). Analysis code is provided in S6 File.

**Table 4:** Radiologists’ accuracy with the presence and absence of AI assistant.

Radiologist	No AI assistance	AI assistance
1	0.777	0.800
2	0.800	1.00
3	0.750	0.650
4	0.750	0.750
Mean	0.769	0.800
SD^*^	0.769	0.147

* SD: Standard Deviation.

**Table 5:** Total interpretation time of radiologists in seconds with and without the AI assistant.

Radiologist	No AI assistance	AI assistance
1	546.0	**444.0**
2	420.3	**308.0**
3	315.6	280.3
4	338.0	**271.0**
Mean	404.9	325.8
SD	104.2	80.3

#### System usability.

**Fig 8:**
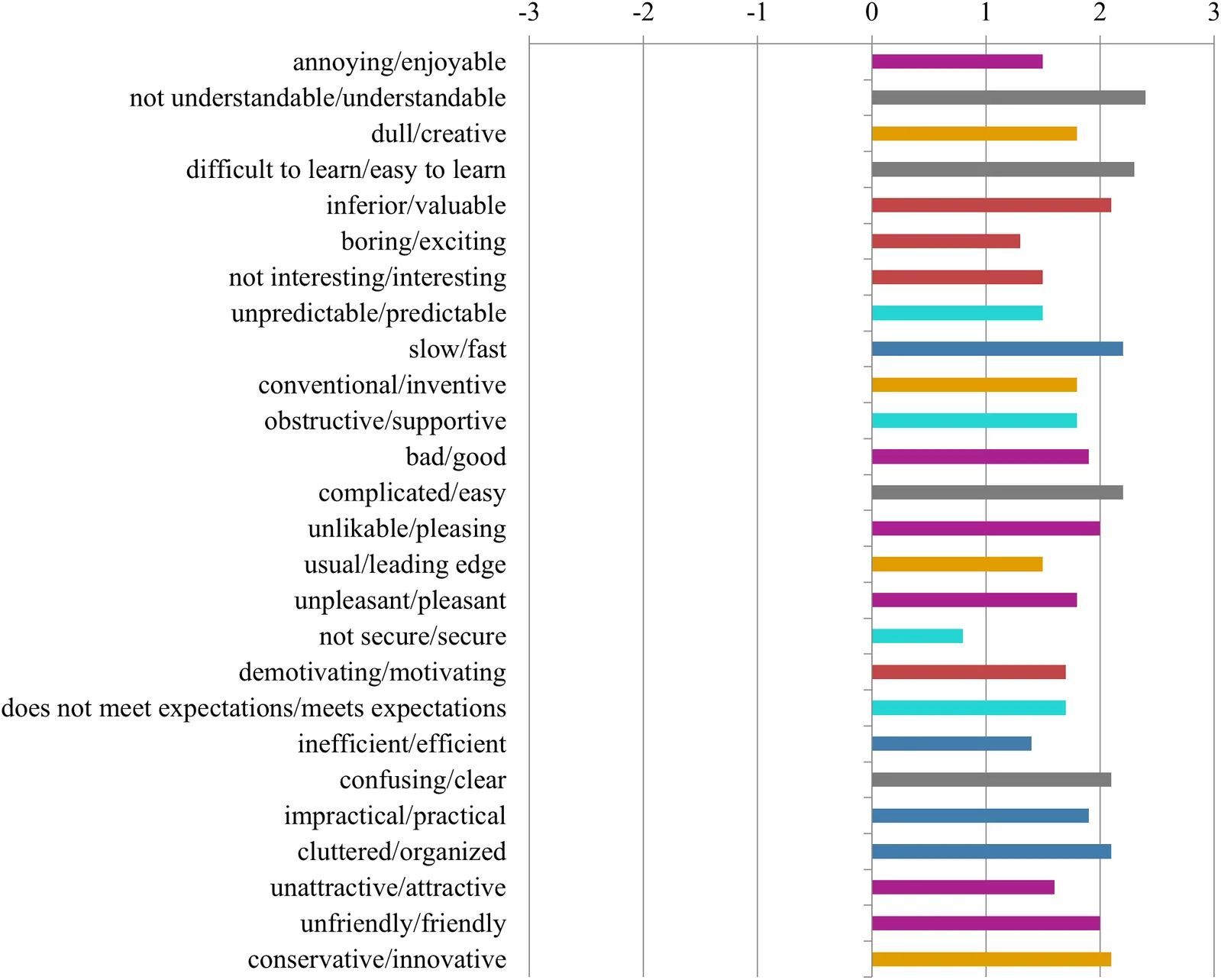
Distribution of average scores given to each system’s attribute by the evaluators. Overall, positive results were obtained, indicating more favorable opinions.

In the second phase, the proposed software was subjected to user experience evaluation. The number of evaluators was calculated utilizing the sample size table provided with the UEQ questionnaire. Considering the intermediate level of agreement between participants and the 95% confidence interval width of 1.15, the sample size was estimated as 10. Therefore, 10 experts in the related fields (six radiologists, one gynecologist, two health informaticians, and one medical physicist) were selected via a convenience sampling method and asked to fill out the UEQ upon complete familiarity with the system’s features and capabilities. To analyze responses, the answered scores were scaled from 1 to 7 to −3 to +3, in which −3 represents the most pessimistic response, 0 is neutral, and +3 is the most optimistic response. Fig 8 depicts the average scores assigned to each attribute by the evaluators. Overall, all scores were in the positive range, mostly between +1 and +2, with a few above +2. The “Secure” attribute obtained the lowest average score with a value of 0.8, and the highest average score was assigned to the “Understandable” attribute with a value of 2.4.

**Fig 9:**
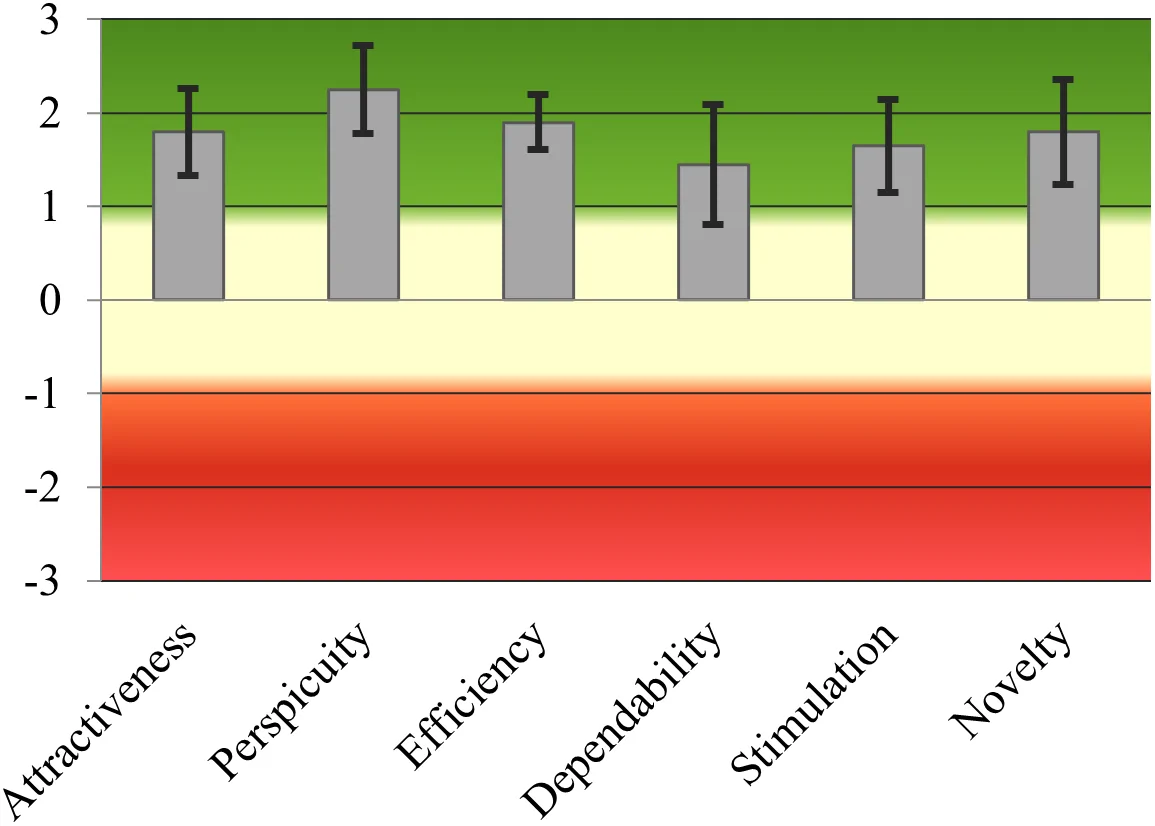
The system’s usability scores across UEQ aspects. This chart includes the mean and confidence interval of user experience ratings for the six evaluation dimensions. The average ratings vary between 1.4 and 2.3, implying acceptable or excellent performance.

Furthermore, the average score related to each evaluation aspect can be calculated via the values of the associated attributes, which are presented in Table 6. Here, a score between −0.8 and 0.8 denotes a more or less neutral assessment of the corresponding aspect, values greater than 0.8 indicate an ideal assessment, and values smaller than −0.8 indicate a poor assessment. Overall, the average scores ranged from 1.4 to 2.3, indicating acceptable to excellent performance of the system. In terms of dependability, the system achieved the lowest score. This criterion further showed the highest SD, which may imply a lack of transparency in some questions associated with dependability for some users, leading to inconsistent responses. Moreover, the highest score belonged to the system’s perspicuity. According to the bar chart in Fig 9, all the average ratings fall within the ideal zone, indicating that the proposed software has received positive feedback from experts across multiple fields.

**Table 6:** The mean, SD, and confidence interval of the scores assigned to each main evaluation aspect of the UEQ.

UEQ aspect	Mean	SD	Width of 95% confidence interval
Attractiveness	1.800	0.749	0.928
Perspicuity	2.250	0.764	0.947
Efficiency	1.900	0.474	0.588
Dependability	1.450	1.039	1.289
Stimulation	1.650	0.801	0.993
Novelty	1.800	0.904	1.120

## Discussion

The contributions in the field of breast cancer detection via mammography image analysis are significant and incorporate a diverse set of models and strategies. The present study proposed a system based on an incremental deep ensemble model with the ability of both synchronous and asynchronous training of multiple CNNs across one or several servers. Utilizing such a model to develop a CAD software has been rarely addressed by previous studies, which was the aim of this research. Deep Learning (DL) models offer significant opportunities for enhancing breast cancer screening, prognosis, diagnosis, and treatment, employing a variety of image modalities such as mammography, ultrasound, Magnetic Resonance Imaging (MRI), and histopathology [54].

The three-tier paradigm was the architecture of choice for our system, which is a ubiquitous pattern in web-based healthcare systems for an extended period [55–60]. As the application expands, additional resources can be allocated to any of the three layers without necessitating modifications to the other ones [61,62]. This flexibility is crucial in the case of varying workloads. Furthermore, this pattern supports the use of different technologies and programming languages for each layer, facilitating optimization and utilization of specialized tools for specific tasks [61]. This paradigm also aids maintenance, as changes to a layer can often be implemented without affecting others, thus reducing downtime and disruption [63,64]. We further tried to integrate the best security practices into the software including authentication and authorization via ASP.NET Identity framework, SSL encrypted communication, password hashing, and reCAPTCHA validation.

The structural and conceptual modeling of this system was carried out using the UML diagrams, which have a broad application for modeling various aspects of healthcare systems [65]. The package diagram revealed the reusability value of encapsulating logical components in the form of DTOs and services. The class diagram exhibited the relationships among the entities and the necessity of a base entity to avoid redundancy. The implementation diagram allowed us to configure the main system components as three servers, distributing the load and promoting scalability via the use of different execution environments (ASP.NET, MSSQL, FastAPI). The sequence diagram illustrated the absence of unnecessary operations in the flow of events and the presence of appropriate feedback in case of errors.

Considering similar commercial systems, the GITAI [41] was one of the few Iranian products similar to ours, which provides risk percentage and lesion localization. Nonetheless, GITAI was developed using a bigger dataset, and its reduction in interpretation delay and sensitivity was higher but with a lower specificity and AUC. Hence, the developers appeared to prioritize maximizing the true positive detection, even at the expense of a higher false positive rate. The Breast-SlimView [42] approached the problem from a different angle by reducing the radiologist’s cognitive load and enabling quick visual inspection of suspicious areas. In contrast, the BINA system generates cancer risk percentage along with saliency maps per mammogram view, indicating the areas worth noting by the radiologist. Furthermore, MammoScreen [25] extends our software capabilities by mammography and DBT analysis, prior-exam comparison, and breast density assessment.

The proposed CAD system yielded a reduction in the radiologist’s interpretation time by nearly 20% while maintaining their accuracy, although the model alone achieved a higher accuracy. This finding may be due to the fact that some users, regardless of the model’s predictions, base the final decision on their own experiences and knowledge, allowing the AI assistant to influence their diagnosis only partially. This issue could stem from the lack of sufficient confidence in such black box tools. The wide SD we report for the AI-assisted accuracy condition can further imply that the trust in AI predictions varies by radiologist. Furthermore, the accuracy values improved with AI assistance. However, this increase was not statistically significant, probably due to the number of readers. Hence, increasing the sample size may render this observation significant. Increased radiologist speed can provide additional health economic benefits, including screening more patients during a standard workday, mitigating radiologist overload, and allowing more time for meticulous review of borderline (e.g., BI-RADS 3–4) cases to reduce misclassifications.

**Fig 10:**
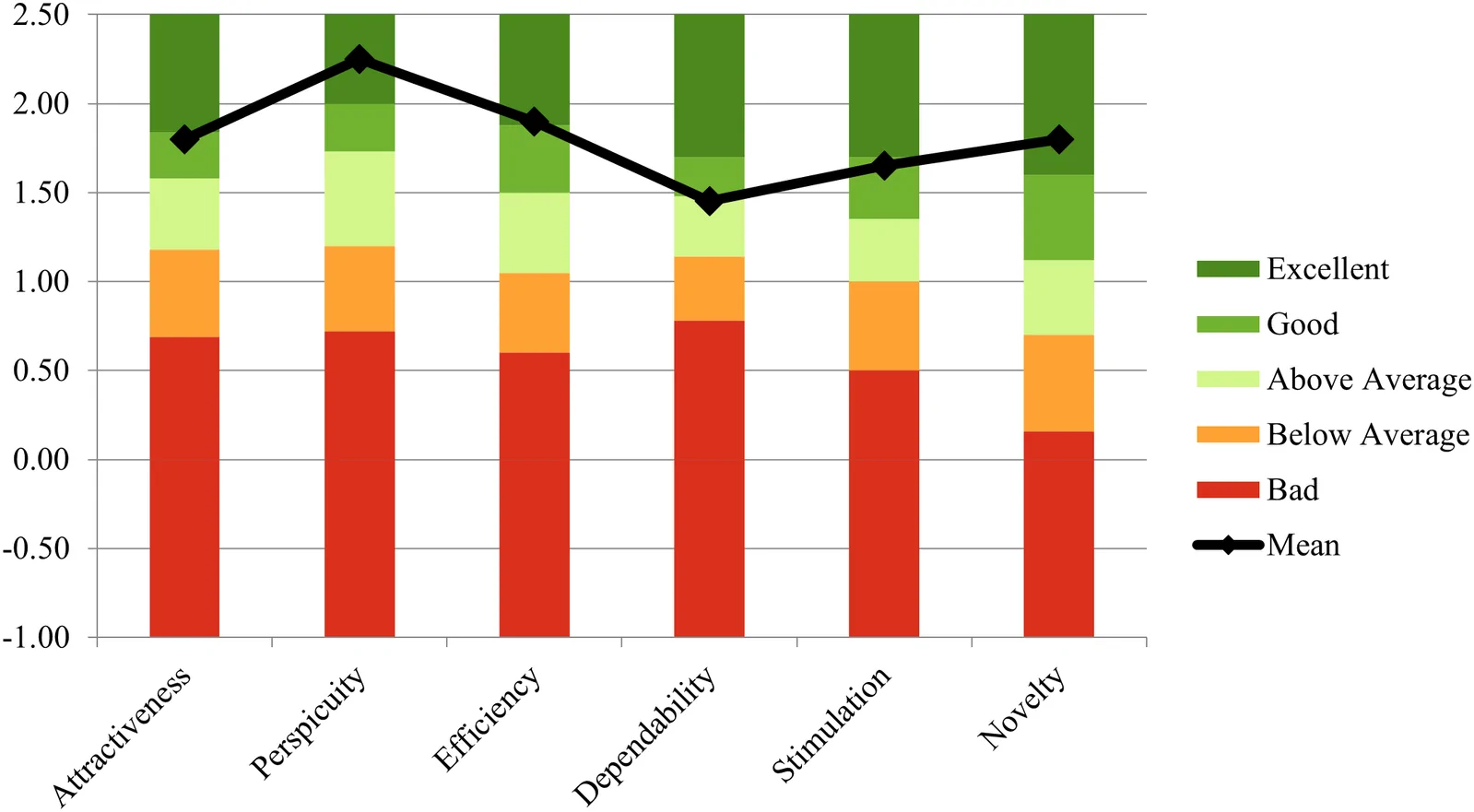
Comparison of the system’s usability against the benchmark. This chart features the average performance scores of the Breast Imaging Novel Assistant (BINA) software in six usability aspects and their position in the UEQ benchmark.

The second part of the findings is dedicated to the user experience evaluation via the UEQ tool. This questionnaire’s format supports immediate user response to express the impressions, feelings, and attitudes gained from using the product. Hence, it has been used for the usability evaluation of a wide range of products, such as a mobile application for early cancer detection [66] and a 3D software for robotic and laparoscopic surgery [67]. The findings indicated high usability of the proposed system across the six UEQ dimensions. Nonetheless, to provide a more precise picture of the product’s quality, it is necessary to compare the acquired scores with those of other established products. Consequently, a benchmark dataset containing the evaluation results of a wide range of products has been issued by the UEQ publisher, which includes 452 products and 20,190 participants [53]. This benchmark categorizes a product as excellent, good, above average, below average, and poor for each of the six aspects. Fig 10 shows the position of the average scores earned by the BINA software among other benchmark products, and Table 7 indicates the meaning of these comparison findings. Accordingly, the proposed system was ranked among the top 10% of products in terms of perspicuity, efficiency, and novelty. In terms of attractiveness and stimulation, 10% of products performed better. And in terms of dependability, 25% of products performed better. The system’s high perspicuity originates from its straightforward user interface, and its high efficiency and novelty can be due to the type of intelligent model and the uniqueness of this system for the experiment population. On the other hand, the lower attractiveness and stimulation towards using the software may indicate insufficient incentives and motivational factors for experts. Finally, the near average dependability may imply the lack of inducing enough sense of security and support for the user’s tasks. Moreover, regarding the probable gradual shift in acquisition protocols, device characteristics, and population demographics, such incremental CAD tools can support continuous alignment with changing environments and promise long-term clinical deployment. In the context of clinical adoption, high perspicuity is crucial for a short learning curve and minimal disruption in the established radiology workflow. Such software reduces cognitive load and promotes seamless integration, thereby supporting faster clinical acceptance. Analogously, strong performance in efficiency and novelty aspects implies the system’s rapid interpretation while offering complementary reports that support the decision-making process. Moreover, since dependability reflects the user’s sense of control and trust, it directly influences clinical adoption. Improving the perceived dependability entails more transparency in the software’s behavior and feedback mechanisms. Therefore, a decent performance across all usability dimensions is critical for clinical reliance on CAD tools. Considering the usability results, our system is suitable for workflow integration, with some room for improvement in dependability, which could be addressed by stating model confidence levels or increasing the interpretability of its decision-making process. However, we should also note the black box nature of the DL models in this matter.

**Table 7:** The performance of the BINA system in comparison with UEQ benchmark data and the interpretation of the scores.

Scale	Mean	Comparison to benchmark	Interpretation
Attractiveness	1.80	Good	10% of results better, 75% of results worse
Perspicuity	2.25	Excellent	In the range of the 10% best results
Efficiency	1.90	Excellent	In the range of the 10% best results
Dependability	1.45	Above Average	25% of results better, 50% of results worse
Stimulation	1.65	Good	10% of results better, 75% of results worse
Novelty	1.80	Excellent	In the range of the 10% best results

The present study encountered some limitations that can be addressed in future efforts. An inaccurate or misleading heatmap can misdirect the radiologist’s attention rather than aiding. Therefore, we recommend quantitative assessment of Grad-CAM++ heatmaps against segmentation masks in the future. Despite the widespread use of three-tier and multi-tier architectures, they have been around for a long period, and more secure and optimized alternatives such as the onion architecture [68] are recommended for future CAD system designs. The presentation of cases during the multi-reader evaluation phase was according to a predefined order. This might have potentially influenced the radiologists’ interpretation time. Therefore, we interpreted the time reduction cautiously and suggest further randomization of cases as a necessity for future work. Another issue in the evaluation phase was the lower number of participating specialists and image samples compared to previous studies. The smaller number of specialists might be due to the lack of sufficient incentives for them to participate, and the smaller number of image samples could be due to the time and energy constraints of the radiologists for interpreting all cases in a single session. Hence, such contributing factors must be addressed and suitable solutions should be considered in future directions. Lastly, further extending data diversity and conducting collaborative cross-institutional evaluations are essential for enhancing the generalizability of such systems.

## Conclusion

Breast imaging analysis has been a renowned field, and ubiquitous CAD systems have been developed and evaluated to facilitate this task progressively. This multi-reader and multi-case research aimed to design, implement, and evaluate a web-based software incorporating a novel incremental model for aiding mammography analysis, which assessed the practicality of a continual learning paradigm in the clinical setting. The proposed system helped increase the reading speed of radiologists while maintaining their diagnostic accuracy. The findings suggested the high usability, especially in terms of transparency, efficiency, and innovation. Therefore, the BINA software potentially enhances breast cancer diagnosis and the decision-making process of specialists, but it still requires further development and validation in broader settings and slight improvements in terms of dependability, which would further strengthen its readiness for clinical integration and interface with existing hospital PACS platforms.

## Supporting information

S1 File Radiologist 1 reading-experiment inputs. Spreadsheet containing the 40 studies, image identifiers, and ViTGEMC outputs presented to radiologist 1.
(XLSX)

S2 File Radiologist 2 reading-experiment inputs. Spreadsheet containing the 40 studies, image identifiers, and ViTGEMC outputs presented to radiologist 2.
(XLSX)

S3 File Radiologist 3 reading-experiment inputs. Spreadsheet containing the 40 studies, image identifiers, and ViTGEMC outputs presented to radiologist 3.
(XLSX)

S4 File Radiologist 4 reading-experiment inputs. Spreadsheet containing the 40 studies, image identifiers, and ViTGEMC outputs presented to radiologist 4.
(XLSX)

S5 File User Experience Questionnaire data analysis workbook. Completed UEQ Data Analysis Tool (version 12) containing the 10 expert responses and derived scale scores.
(XLSX)

S6 File Multi-reader experiment analysis notebook. Python notebook used to compute diagnostic accuracy and interpretation-time statistics from the radiologist reading experiment.
(DOCX)
