# Supplementary material for: Development and evaluation of a deep incremental learning system for mammography image analysis
Source: PLoS One. 2026 Sep 25;21(9):e0358861. doi: 10.1371/journal.pone.0358861 (PMC13614561; doi:10.1371/journal.pone.0358861)
Supplement: Supplementary file 1 [file pone.0358861.s006.docx]

import pandas as pd

import sys

sys.path.append(r"D:\Python training\My modules")

from utils import scantree, get_metrics

from sklearn.metrics import confusion_matrix, roc_curve, auc

def get_metrics(targets, preds):

metrics = {}

confusion_mtx = confusion_matrix(targets, preds)

TN = confusion_mtx[0][0]

FN = confusion_mtx[1][0]

TP = confusion_mtx[1][1]

FP = confusion_mtx[0][1]

metrics["acc"] = (TN + TP) / (TN + TP + FN + FP)

metrics["recall_sen"] = TP / (TP + FN)

metrics["spc"] = TN / (TN + FP)

metrics["prc_ppv"] = TP / (TP + FP)

metrics["npv"] = TN / (TN + FN)

metrics["f1"] = (2 * metrics["prc_ppv"] * metrics["recall_sen"]) / (metrics["prc_ppv"] + metrics["recall_sen"])

fpr, tpr, thresholds = roc_curve(targets, preds)

metrics["roc_auc"] = auc(fpr, tpr)

return metrics

def birads_to_label(birads):

if birads in ['1', '2', '3', '4a']:

return 0

elif birads in ['4b', '4c', '5', '6']:

return 1

def str_to_label(string):

if string in ['0', '1', '19a']:

return 0

elif string in ['19', '19b', '19c', '2']:

return 1

all_imgs_paths = list(scantree(".", ".png")) + list(scantree(".", ".dcm")) + list(scantree(".", ".dicom"))

\# all_imgs_paths = list(filter(lambda p : ".png" in p, all_imgs_paths))

\# all_imgs_paths

import os

def human_performace_results(database_images, database_studies, database_modelOutputs):

human_labels_noAI = []

human_labels_AI = []

real_labels_noAI = []

real_labels_AI = []

AI_labels = []

for i, study in database_studies.iterrows():

images = database_images[database_images["StudyId"] == study["Id"]]

assert len(images) == 2

for j, image in images.iterrows():

dcmPath = image["DICOMPath"]

dcmName_segs = os.path.splitext(dcmPath)[0].replace("studies/", "").split("/")

org_paths = list(filter(lambda x : all([seg in x for seg in dcmName_segs]), all_imgs_paths))

org_paths = list(filter(lambda p : "seperate images" not in p, org_paths))

labels = []

for path in org_paths:

label_indx = path.find("label") + len("label ")

end = path.find("\\", label_indx)

labels.append(path[label_indx:end])

if len(labels) == 0:

print(dcmPath)

continue ################### Find the labels ############### -> Missing images were found and added to the corresponding folders

assert all([labels[0] == label for label in labels])

human_label = birads_to_label(str(study["BIRADS"]))

if human_label == None:

continue

real_label = str_to_label(labels[0])

model_output = database_modelOutputs[database_modelOutputs["ImageId"] == image["Id"]]

if len(model_output) == 0: # There is no AI evaluation

human_labels_noAI.append(human_label)

real_labels_noAI.append(real_label)

else:

assert len(model_output) == 1

human_labels_AI.append(human_label)

real_labels_AI.append(real_label)

AI_labels.append(int(model_output.iloc[0]["Label"] == "Malignant"))

confusion_mtx = confusion_matrix(real_labels_noAI, human_labels_noAI)

TN = confusion_mtx[0][0]

FN = confusion_mtx[1][0]

TP = confusion_mtx[1][1]

FP = confusion_mtx[0][1]

print("TN, FN, TP, FP:", TN, FN, TP, FP)

print("Human without AI metrics:", get_metrics(real_labels_noAI, human_labels_noAI))

print("Human with AI metrics:", get_metrics(real_labels_AI, human_labels_AI))

print("AI metrics:", get_metrics(real_labels_AI, AI_labels))

print("AUCs:",

roc_auc_score(real_labels_noAI, human_labels_noAI, average = 'micro'),

roc_auc_score(real_labels_AI, human_labels_AI, average = 'micro'),

roc_auc_score(real_labels_AI, AI_labels, average = 'micro'))

drahmadinejad_studies = pd.read_excel("DrAhmadinejad.xlsx", sheet_name = "Studies")

drahmadinejad_images = pd.read_excel("DrAhmadinejad.xlsx", sheet_name = "Images")

drahmadinejad_modelOuts = pd.read_excel("DrAhmadinejad.xlsx", sheet_name = "ModelOutputs")

human_performace_results(drahmadinejad_images, drahmadinejad_studies, drahmadinejad_modelOuts)

TN, FN, TP, FP: 18 6 14 2

Human without AI metrics: {'acc': 0.8, 'recall_sen': 0.7, 'spc': 0.9, 'prc_ppv': 0.875, 'npv': 0.75, 'f1': 0.7777777777777777, 'roc_auc': 0.8}

Human with AI metrics: {'acc': 1.0, 'recall_sen': 1.0, 'spc': 1.0, 'prc_ppv': 1.0, 'npv': 1.0, 'f1': 1.0, 'roc_auc': 1.0}

AI metrics: {'acc': 0.9, 'recall_sen': 0.8, 'spc': 1.0, 'prc_ppv': 1.0, 'npv': 0.8333333333333334, 'f1': 0.888888888888889, 'roc_auc': 0.9}

AUCs: 0.8 1.0 0.9

for results_file in ["DrAhmadi.xlsx", "DrAhmadinejad.xlsx", "DrMirzaie.xlsx", "DrShayegh.xlsx"]:

print("#"*10, results_file, "#"*10)

studies = pd.read_excel(results_file, sheet_name = "Studies")

images = pd.read_excel(results_file, sheet_name = "Images")

modelOuts = pd.read_excel(results_file, sheet_name = "ModelOutputs")

human_performace_results(images, studies, modelOuts)

print("\n")

########## DrAhmadi.xlsx ##########

TN, FN, TP, FP: 16 6 12 2

Human without AI metrics: {'acc': 0.7777777777777778, 'recall_sen': 0.6666666666666666, 'spc': 0.8888888888888888, 'prc_ppv': 0.8571428571428571, 'npv': 0.7272727272727273, 'f1': 0.75, 'roc_auc': 0.7777777777777777}

Human with AI metrics: {'acc': 0.8, 'recall_sen': 0.6, 'spc': 1.0, 'prc_ppv': 1.0, 'npv': 0.7142857142857143, 'f1': 0.7499999999999999, 'roc_auc': 0.8}

AI metrics: {'acc': 0.9, 'recall_sen': 0.8, 'spc': 1.0, 'prc_ppv': 1.0, 'npv': 0.8333333333333334, 'f1': 0.888888888888889, 'roc_auc': 0.9}

AUCs: 0.7777777777777777 0.8 0.9

########## DrAhmadinejad.xlsx ##########

TN, FN, TP, FP: 18 6 14 2

Human without AI metrics: {'acc': 0.8, 'recall_sen': 0.7, 'spc': 0.9, 'prc_ppv': 0.875, 'npv': 0.75, 'f1': 0.7777777777777777, 'roc_auc': 0.8}

Human with AI metrics: {'acc': 1.0, 'recall_sen': 1.0, 'spc': 1.0, 'prc_ppv': 1.0, 'npv': 1.0, 'f1': 1.0, 'roc_auc': 1.0}

AI metrics: {'acc': 0.9, 'recall_sen': 0.8, 'spc': 1.0, 'prc_ppv': 1.0, 'npv': 0.8333333333333334, 'f1': 0.888888888888889, 'roc_auc': 0.9}

AUCs: 0.8 1.0 0.9

########## DrMirzaie.xlsx ##########

TN, FN, TP, FP: 20 10 10 0

Human without AI metrics: {'acc': 0.75, 'recall_sen': 0.5, 'spc': 1.0, 'prc_ppv': 1.0, 'npv': 0.6666666666666666, 'f1': 0.6666666666666666, 'roc_auc': 0.75}

Human with AI metrics: {'acc': 0.65, 'recall_sen': 0.3, 'spc': 1.0, 'prc_ppv': 1.0, 'npv': 0.5882352941176471, 'f1': 0.4615384615384615, 'roc_auc': 0.65}

AI metrics: {'acc': 0.9, 'recall_sen': 0.8, 'spc': 1.0, 'prc_ppv': 1.0, 'npv': 0.8333333333333334, 'f1': 0.888888888888889, 'roc_auc': 0.9}

AUCs: 0.75 0.65 0.9

########## DrShayegh.xlsx ##########

TN, FN, TP, FP: 18 8 12 2

Human without AI metrics: {'acc': 0.75, 'recall_sen': 0.6, 'spc': 0.9, 'prc_ppv': 0.8571428571428571, 'npv': 0.6923076923076923, 'f1': 0.7058823529411764, 'roc_auc': 0.7500000000000001}

Human with AI metrics: {'acc': 0.75, 'recall_sen': 0.5, 'spc': 1.0, 'prc_ppv': 1.0, 'npv': 0.6666666666666666, 'f1': 0.6666666666666666, 'roc_auc': 0.75}

AI metrics: {'acc': 0.9, 'recall_sen': 0.8, 'spc': 1.0, 'prc_ppv': 1.0, 'npv': 0.8333333333333334, 'f1': 0.888888888888889, 'roc_auc': 0.9}

AUCs: 0.7500000000000001 0.75 0.9
